# Temporal cell wall changes during cold acclimation and deacclimation and their potential involvement in freezing tolerance and growth

**DOI:** 10.1111/ppl.13837

**Published:** 2022-12-29

**Authors:** Tatsuya Kutsuno, Sushan Chowhan, Toshihisa Kotake, Daisuke Takahashi

**Affiliations:** ^1^ Graduate School of Science & Engineering Saitama University Saitama Japan

## Abstract

Plants adapt to freezing stress through cold acclimation, which is induced by nonfreezing low temperatures and accompanied by growth arrest. A later increase in temperature after cold acclimation leads to rapid loss of freezing tolerance and growth resumption, a process called deacclimation. Appropriate regulation of the trade‐off between freezing tolerance and growth is necessary for efficient plant development in a changing environment. The cell wall, which mainly consists of polysaccharide polymers, is involved in both freezing tolerance and growth. Still, it is unclear how the balance between freezing tolerance and growth is affected during cold acclimation and deacclimation by the changes in cell wall structure and what role is played by its monosaccharide composition. Therefore, to elucidate the regulatory mechanisms controlling freezing tolerance and growth during cold acclimation and deacclimation, we investigated cell wall changes in detail by sequential fractionation and monosaccharide composition analysis in the model plant *Arabidopsis thaliana*, for which a plethora of information and mutant lines are available. We found that arabinogalactan proteins and pectic galactan changed in close coordination with changes in freezing tolerance and growth during cold acclimation and deacclimation. On the other hand, arabinan and xyloglucan did not return to nonacclimation levels after deacclimation but stabilized at cold acclimation levels. This indicates that deacclimation does not completely restore cell wall composition to the nonacclimated state but rather changes it to a specific novel composition that is probably a consequence of the loss of freezing tolerance and provides conditions for growth resumption.

## 1 INTRODUCTION

Because plants are immobile organisms, they cannot avoid stresses caused by environmental changes. Plants exposed to freezing stress are known to suffer irreversible damage to the plasma membranes as a combined effect of dehydration, physical pressure, and hyperosmotic stress due to the expansion of extracellular ice crystals (Steponkus, 1984). Many temperate plants can improve their freezing tolerance through a mechanism called cold acclimation (CA, Thomashow, 1999). CA, which occurs when plants are exposed to nonfreezing low temperatures, is often accompanied by growth arrest (Levitt, 1980), and can reduce freezing injuries through various changes in transcript, protein, and metabolite levels (Hannah et al., 2005; Kaplan et al., 2006; Shi et al., 2018; Takahashi et al., 2013).

On the other hand, a process called cold deacclimation (DA), in which plants lose their acquired freezing tolerance and resume growth upon sensing an increase in temperature after CA, has recently attracted attention (Pagter & Arora, 2013; Vyse et al., 2019). The kinetics of DA at the transcriptome level is extremely rapid, with most changes already occurring in the first 24 h (Oono et al., 2006; Pagter et al., 2017). In many studies focusing on mRNA expression levels, most changes induced by DA are inverse responses to changes induced by CA (Nakaminami et al., 2014; Oono et al., 2006; Pagter et al., 2017). Proteomic analyses investigating the effects of DA on plasma membrane proteins have shown that many plasma membrane proteins that increase or decrease during CA show opposite behavior during DA (Miki et al., 2018). However, it is speculated that there are complex processes that cannot be described simply as the inverses of certain CA reactions, especially in some water‐soluble metabolites (Zuther et al., 2015).

The cell wall is the site of ice crystal formation during the freezing process, and the cell wall can prevent extracellular freezing damage to the plasma membrane (Panter et al., 2020). At the same time, it is well known that cell wall stress, along with turgor pressure, regulates cell expansion and growth (Cosgrove, 2005). Given that the availability of carbon resources needed for acquisition of freezing tolerance or growth is limited, and the distribution of these resources is important, there is a trade‐off between freezing tolerance and growth rate (Savage & Cavender‐Bares, 2013; Wingler, 2015). Therefore, the cell wall, which is involved in both freezing tolerance and growth, is expected to be an important element in regulating the balance between the two.

The primary cell wall consists of cellulose microfibrils forming a strong reinforcement structure, with hemicellulose linking them together and pectin filling the gaps between microfibrils (Cosgrove, 2022). Pectin and hemicellulose can be sequentially extracted as chelate‐ (e.g. EDTA) and alkaline (e.g. KOH) ‐soluble components by taking advantage of their properties as cell wall‐filling materials by gelation with calcium crosslinks and their strong hydrogen bonding to cellulose microfibrils, respectively, and finally, crystalline cellulose remains as a precipitate (Zablackis et al., 1995). Heavily glycosylated proteins, as represented by arabinogalactan proteins (AGPs) are also present in the cell wall and play roles as information molecules, cell adhesion factors, and regulators of cell wall synthesis (Lopez‐Hernandez et al., 2020; Shi et al., 2003; Willats & Knox, 1996; Yoshimi et al., 2020).

In Arabidopsis, the pectin cross‐link structure and hemicellulose modification affect basal freezing tolerance (Panter et al., 2019; Shi et al., 2014). In pea, wheat, *Allium fistulosum*, and Arabidopsis, changes in pectin and hemicellulose during CA are assumed to lead to a finer cell wall mesh and cell wall strengthening, contributing to acquired freezing tolerance (Baldwin et al., 2014; Chen et al., 2018; Liu et al., 2022; Takahashi et al., 2019; 2020; Willick et al., 2018). Although many functional aspects of AGPs in freezing tolerance remain unexplored, it has been shown that the expression of some AGPs is increased upon cold treatment in wheat (Faik et al., 2006). Therefore, the structural and compositional changes of the cell wall during CA and DA would seem to be important in the acquisition and/or loss of freezing tolerance.

Changes in cell wall composition and structure, particularly hemicellulose modifications, are thought to affect mechanical linkage (Albersheim et al., 2010) and/or the self‐assembly of cellulose microfibrils (Cosgrove, 2022). These are believed to result in cell wall loosening and thereby cause cell expansion and growth (Zhang et al., 2021). How pectin regulates cell growth is not yet clearly understood, but it has recently been proposed that the insertion of pectin into the cellulose microfibrils is a necessary process for irreversible cell wall expansion (Cosgrove, 2022). Given the above, it may well be that in CA and DA processes, where growth characteristics change dynamically, cell wall changes contribute to freezing tolerance as well as to growth control in response to temperature changes.

Thus, strict control of the balance between the acquisition of freezing tolerance and growth rate is important during the freezing adaptation process in CA and the growth resumption process in DA. Since the cell wall is involved in both freezing tolerance and growth, investigating cell wall changes in CA and DA will lead to a better understanding of the mechanisms that control freezing tolerance and growth in a changing environment. Therefore, we performed an integrated compositional analysis of cell wall polysaccharides in *Arabidopsis thaliana*. The obtained data on cell wall monosaccharide composition provides the first clue to finding the relevant polysaccharide molecular species and structures in CA and DA, leading to the elucidation of molecular mechanisms using suitable Arabidopsis mutants. This study provides a basis for elucidating the significance of molecular cell wall changes concerning the regulation of freezing tolerance and growth in CA and DA.

## 2 MATERIALS AND METHODS

### 2.1 Growth conditions of *Arabidopsis thaliana*

**FIGURE 1:**
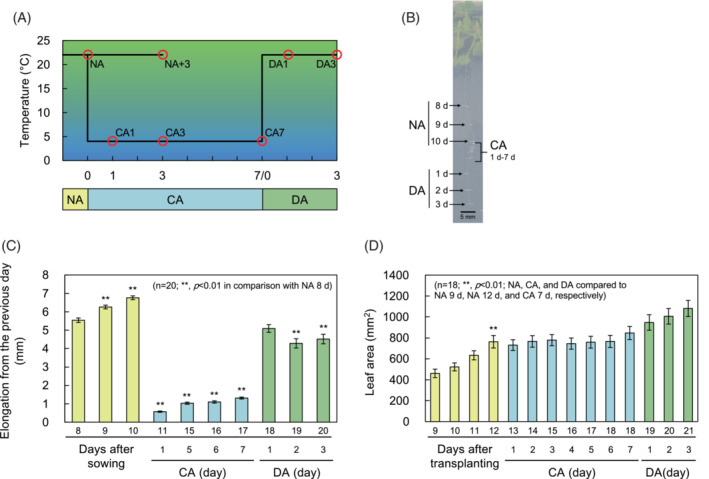
Acclimation scheme and physiological changes in Arabidopsis plants. (A) Plants grown at room temperature with fully expanded leaves were designated as NA, and those transferred to 4°C for 1, 3, and 7 days were designated as CA1, 3, and 7. The plants that were then returned to room temperature for 1 and 3 days were designated DA1 and 3. (B) Changes in root elongation due to cold acclimation (CA) and cold deacclimation (DA). (C) Root elongation was calculated by comparing it to the length of the root the day before. Error bars indicate ± SEM (*n* = 20). (D) Plants were photographed, and leaf area was measured daily to monitor growth in the above ground part of the plant. Error bars indicate ± SEM (*n* = 18). Significant differences (Dunnett's test) are indicated by asterisks above the bars (*p < 0.05, **p < 0.01).

Arabidopsis (*A. thaliana*) Columbia‐0 plants were grown on Murashige‐Skoog (MS) agar medium at 22°C under light and dark conditions (16/8 h light/dark, 120 μmol m^−2^ s^−1^) for 7 days and then transplanted to mineral wool (Nittobo). These plants were grown for an additional 14 days under the same temperature and light conditions and designated as NA (nonacclimated) plants (Figure 1A). NA plants that were further grown at 4°C under light and dark conditions (12/12 h light/dark, 120 μmol m^−2^ s^−1^) for 1, 3, and 7 days were designated CA1, CA3, and CA7, respectively (Figure 1A). Since Arabidopsis acquires maximum freezing tolerance after 7 days at 4°C (Uemura et al., 1995), fully cold acclimated CA7 plants were used for DA treatments. DA treatments were performed by transferring CA7 plants to 22°C for 1 and 3 days, designated DA1 and DA3, respectively (Figure 1A). Three days of DA is sufficient time to return to NA levels of freezing tolerance (Miki et al., 2018; Vyse et al., 2022). In parallel with the CA treatment, plants grown for 3 days under NA conditions from NA plants were designated as NA+3 (Figure 1A) and used as controls to be compared with DA plants. The aboveground parts of these plants were used to evaluate freezing tolerance, cell wall analysis, and osmolarity measurements. Unless otherwise noted, the number of replicates was three.

### 2.2 Root elongation and leaf area measurement

Plants used for root elongation measurements were sown on MS agar medium and grown for 10 days with the plate upright under NA conditions. These plates were then transferred to CA conditions for 7 days and grown under DA conditions for 3 days. Photographs of the roots were taken each day and root length was measured by Image J to record the growth compared to the previous day. The number of replicates was 20.

Plants for leaf area measurements were grown on MS agar medium for 7 days as described in Section 3.1, transplanted to mineral wool and grown for an additional 12 days under NA conditions. Subsequently, CA treatment was performed for 7 days, followed by a 3‐day DA treatment. Photographs of the above‐ground parts were taken daily, and the leaf area was measured using Image J. The number of replicates was 18.

### 2.3 Measurement of freezing tolerance and osmolarity

The experiment was based on the methods of Thalhammer et al. (2014) and Uemura et al. (1995). The test tubes were filled with 200 μl of water, and above‐ground parts of the plants placed so the hypocotyls were immersed in the water. The test tubes were then cooled in a programmed freezer. First, they were incubated at −2°C for 30 min, after which ice particles were added, and incubation continued for 60 min. The temperature was then lowered at a rate of 2°C per h. At −4, −6, −8, −10, −12, and −16°C, samples were transferred to 4°C and thawed for 12 h in dark. Three ml of water was added, and the samples were shaken for 2 h. After measuring electrical conductivity, the samples were boiｗled for 15 min and the electrical conductivity was measured again. The degree of freezing injury was determined from the ratio of the electrolyte leakage before and after boiling. Freezing tolerance was evaluated at LT_50_, the temperature (°C) at which the electrolyte leakage was 50%. The equation of the survival curve was determined by Rodbard, a curve fitting function provided by ImageJ software (ver. 2.1.0). LT_50_ was calculated based on the equation.

For osmolarity measurements, plants were ground with a pestle and centrifuged at 21,500 *g* for 5 min at 4°C. The tissue sap was collected, heated at 95°C for 10 min and centrifuged again. The supernatant was collected and osmolarity was measured with a freezing point depression osmometer (Type‐13 DR micro‐osmometer, Roebling). The number of replicates in the above experiments was three.

### 2.4 Fractionation and quantification of cell wall polysaccharides

Aboveground parts of the plants were harvested and ground in a mortar. The homogenized samples were collected in microtubes and centrifuged at 21,500 *g* for 5 min at 4°C. The supernatant was used as the soluble fraction. The precipitate was suspended in 800 μl of 80% ethanol and boiled for 2 min. The mixture was then centrifuged at 21,500 *g* for 5 min at 4°C. The supernatant was discarded, and the pellet was resuspended in 500 μl water. Then, 500 μl of amylase reaction solution (50 mM MOPS‐KOH, pH 6.5, 0.04 units α‐amylase) was added, and the samples incubated at 37°C for 2 h. After centrifugation, the supernatant was collected as the starch fraction. The precipitate was suspended in 500 μl water, boiled for 10 min and centrifuged at 21,500 *g* for 5 min at 4°C. The supernatant was collected as the hot water fraction. This step was repeated twice. Thereafter, the same procedures were performed with 50 mM EDTA (pH 8.0)/50 mM sodium phosphate buffer (pH 6.8) and 4 M KOH supplemented with 10.6 mM NaBH_4_ and the supernatants at each step were collected as the EDTA and KOH fractions. The KOH fraction was neutralized by the addition of glacial acetic acid. The precipitate was washed with water, ethanol, and diethyl ether and air‐dried overnight. The resultant powder was hydrolyzed in 100 μl of 72% sulfuric acid for 1 h, then in 800 μl of 8% sulfuric acid for 4 h and collected as the cellulose fraction. The above fractionation methods are summarized in Figure S1. The total sugar content or sugar concentration in all subsequent experiments was determined by the phenol‐sulfuric acid method (DuBois et al., 1956).

AGP content was determined by radial gel diffusion assay (van Holst & Clarke, 1985). The soluble fraction (2 μl) was applied to a gel plate containing 0.004% (w/v) β‐glucosyl‐Yariv reagent, 75 mM NaCl, 0.01% (w/v) sodium azide, and 1% (w/v) agarose. The area of the emerging halo was measured by Image J and quantified using gum arabic as a standard.

### 2.5 Sugar composition analysis by HPAEC‐PAD

Fractionated samples were dialyzed against water for 2 days and lyophilized. A 50 μg of sugar was aliquoted and adjusted with water to a volume of 100 μl. Then 100 μl of 4 N trifluoroacetic acid (TFA) was added to adjust the final concentration to 2 N. The polysaccharides were hydrolyzed by heating in an autoclave for 60 min at 120°C. Then, to remove TFA from the hydrolyzed samples, drying by Speed Vac (TAITEC VC‐36R) and redissolving in water were repeated three times. The resultant hydrolysate was dissolved in 250 μl of water. Fifty μl of sample was subjected to high‐performance anion exchange chromatography‐pulsed amperometric detection (HPAEC‐PAD) with the ICS‐5000^+^series system equipped with a CarboPac PA‐1 column (Thermo Fisher Scientific). Elution was carried out using distilled water, 0.1 M sodium hydroxide with 0.5 M sodium acetate at a flow rate of 1.0 ml min^−1^ as described previously (Ishikawa et al., 2000).

### 2.6 Statistical analysis

Statistical significance of differences was determined by Dunnett's test for multiple comparisons at the *p* < 0.05 level using the R software. The data from monosaccharide analysis was Pareto‐scaled for principal component analysis (PCA) using the *prcomp* function in R.

## 3 RESULTS

### 3.1 Effects of CA and DA treatment on growth and freezing tolerance

In this study, root growth rate and leaf area, which are plant growth indicators, were measured continuously to examine the effects of CA and DA in the laboratory growth environment (Figure 1). Whereas the mean daily root elongation of CA plants was much smaller than that of NA plants, the growth significantly recovered in DA plants (6.2 mm in NA, 1.0 mm in CA, and 4.6 mm in DA, Figure 1B,C). Similar to what was observed for root elongation, DA plants showed an increase in leaf area each day, whereas CA plants showed no change (Figure 1D). This indicates that, unlike CA plants, DA‐treated plants have growth characteristics similar to NA plants.

**FIGURE 2:**
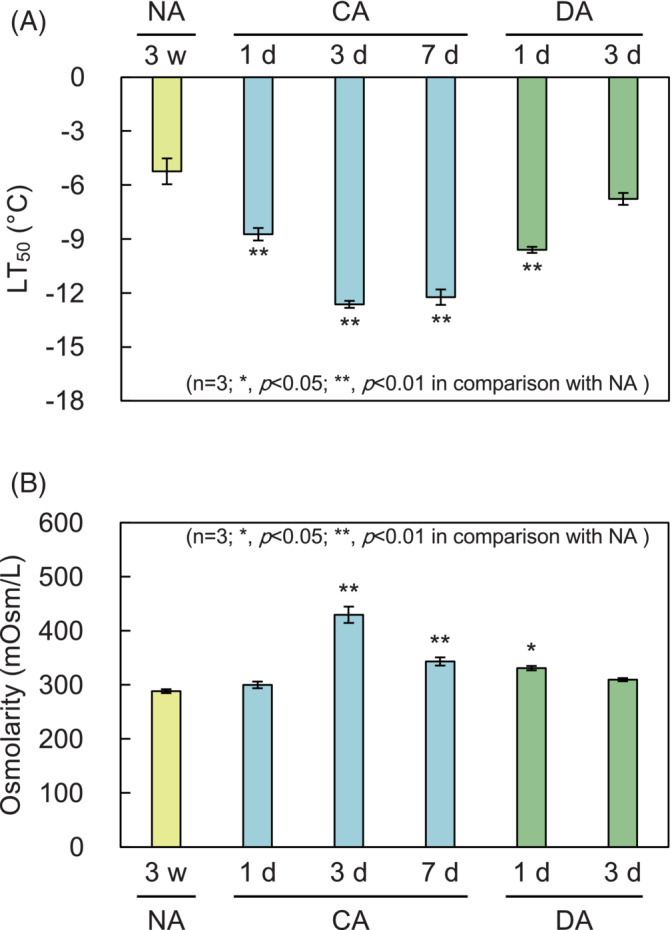
Changes in freezing tolerance and osmotic concentration during the acclimation process. (A) Freezing tolerance was measured by electrolyte leakage assay. Values represent the temperature at which 50% of the electrolyte leaked out (LT_50_). (B) Osmotic concentration (mOsm/L) during the acclimation process was measured by the freezing point depression method. Error bars indicate ± SEM (*n* = 3). Significant differences (Dunnett's test) between non acclimation (NA) and other treatment samples are indicated by asterisks above the bars of cold acclimation (CA) or cold deacclimation (DA) samples, respectively (*p < 0.05, **p < 0.01).

Freezing tolerance was evaluated using the electrolyte leakage method (Figure 2A). The temperature at which electrolyte leakage from tissues reached 50% (LT_50_) in NA was −5.4°C. CA plants showed a significant difference from NA plants, with LT_50_ reaching a minimum of −12.5°C in CA3. In DA, the LT_50_ went back up to −6.8°C at DA3, which was not significantly different from NA. The best‐known intracellular response for improving freezing tolerance in plants is lowering freezing point temperature through accumulating soluble sugars and compatible solutes (Koster & Lynch, 1992). Therefore, we also investigated the osmotic concentration during CA and DA (Figure 2B). Osmotic concentration was maximal for CA3 plants and was significantly lower for DA3 than for CA3 plants. These observations demonstrate that freezing tolerance increased in CA and decreased in DA under the environmental conditions of this study as soluble compounds increased and decreased (Figure 2A).

### 3.2 Cell wall fractionation

**FIGURE 3:**
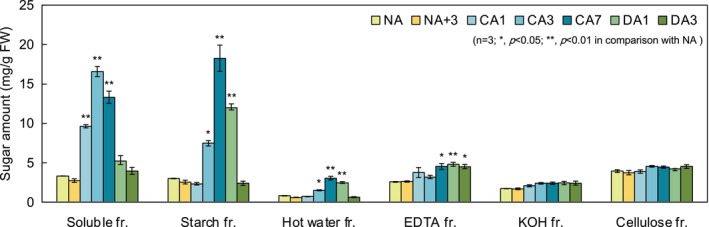
Comparison of proportions of total sugar content fractions. Values represent the content of sugar per gram fresh weight. Error bars indicate ± SEM (*n* = 3). Significant differences (Dunnett's test) between non acclimation (NA) and other treatment samples are indicated by asterisks above the bars of NA+3, cold acclimation (CA) or cold deacclimation (DA) samples, respectively (*p < 0.05, **p < 0.01).

Next, to understand the relevance of the cell wall in these processes, we fractionated the sugars contained within the tissues and analyzed their compositional changes in detail (Figure S1). Looking at the sugar content per fresh weight (FW) of each fraction (Figure 3), soluble sugars and starch increased with the progression of CA and decreased sharply with DA. The other four fractions all contained cell wall polysaccharides. Pectin (the EDTA fraction) was significantly increased by CA. Hemicellulose (the KOH fraction) also tended to increase during CA. The overall percentage of each cell wall‐derived fraction did not change significantly with CA or DA (Figure S2). However, the hot water fraction was exceptional because it was affected by the starch fraction in the sequential fractionation. In fact, Pearson's correlation coefficient indicated that the amount of Glc in the hot water fraction was highly correlated with the amount of starch fraction (*r* = 0.96, Table S1).

### 3.3 Sugar composition analysis

Soluble sugars and the cell wall are composed of various monosaccharides. Therefore, we analyzed the proportions of monosaccharides in the fractions by HPAEC‐PAD, except for the starch and cellulose fractions, which consist mostly of starch and cellulose, respectively, and the hot water fraction, which is strongly affected by the starch fraction in the sequential fractionation.

**FIGURE 4:**
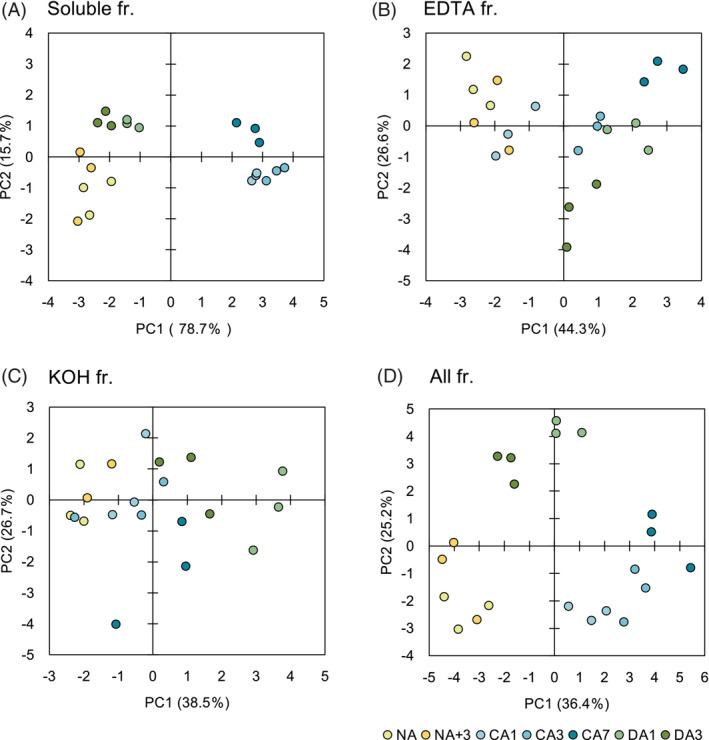
Principal component analysis (PCA) of monosaccharide composition in (A) soluble, (B) EDTA, (C) KOH, and (D) all fractions. Each fraction obtained from non acclimation (NA), cold acclimation (CA), and cold deacclimation (DA) leaves was subjected to HPAEC‐PAD and calculated content of each monosaccharide per gram fresh weight was further analyzed by PCA. Score plots are shown for PC1 and PC2. The fraction of the total variance explained by each PC is indicated in parenthesis.

A PCA was performed to determine the global trend in monosaccharide composition of each fraction (Figure 4). In the soluble fraction, comprised mainly of soluble sugars, the NA and DA clusters were completely separated from the CA clusters (Figure 4A). This implies that CA is causing dramatic accumulation and compositional changes in soluble sugars. On the other hand, in the EDTA and KOH fractions (Figure 4B,C), the NA samples tended to form different clusters upon CA, but the clusters moved in a different direction from NA upon DA. When trends in compositional data for these three fractions were integrated (Figure 4D), they formed three separate clusters: NA, CA, and DA.

**FIGURE 5:**
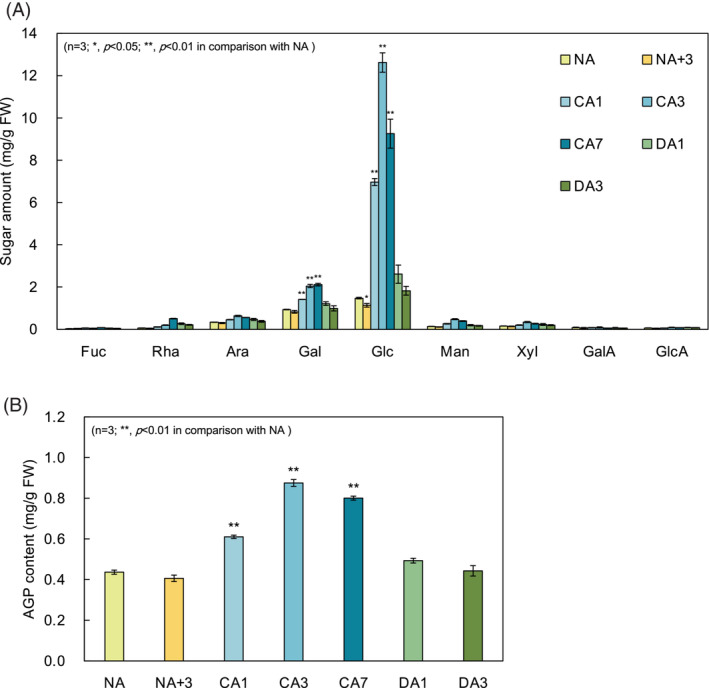
Characterizations of soluble fractions extracted from non acclimation (NA), cold acclimation (CA), and cold deacclimation (DA) leaves. (A) Monosaccharide composition of soluble fractions determined by high‐performance anion exchange chromatography‐pulsed amperometric detection. Values represent the amount of each monosaccharide per gram fresh weight. (B) Arabinogalactan protein content in soluble fraction estimated by radial gel diffusion assay with Yariv reagent. Values represent the equivalent amount of gum arabic. Error bars indicate ± SEM (*n* = 3). Significant differences (Dunnett's test) between NA and other treatment samples are indicated by asterisks above the bars of NA+3, CA or DA samples, respectively (*p < 0.05, **p < 0.01). Fuc, fucose; GalA, galacturonic acid; GlcA, glucuronic acid; Man, mannose

Sugar composition analysis of the soluble fraction by HPAEC‐PAD (Figure 5A) showed that CA caused a marked increase and DA a decrease in various sugars, including d‐glucose (Glc), l‐arabinose (Ara), and d‐galactose (Gal). The change in Glc was particularly pronounced, reflecting the increase in soluble monosaccharides, which is the most prominent change during CA. On the other hand, there were noticeable changes in Ara and Gal. Although Ara and Gal are present in the pectin structure, it can be assumed that they did not derive from water‐soluble pectin, since Rha and GalA, which constitute the main chain of pectin, were almost absent in the soluble fraction. Because these two monosaccharides showed correlated changes, we concluded that they originated from identical molecules, and thus originated from the sugar chains of AGP, a representative proteoglycan of plant cells and is predominantly extracted in the water‐soluble fraction. This is strongly supported by the fact that the quantity of AGP content calculated using Yariv reagent, a reagent that binds specifically to AGP (Kitazawa et al., 2013), was consistent with the results of the sugar composition analysis in Figure 5A (Figure 5B).

**FIGURE 6:**
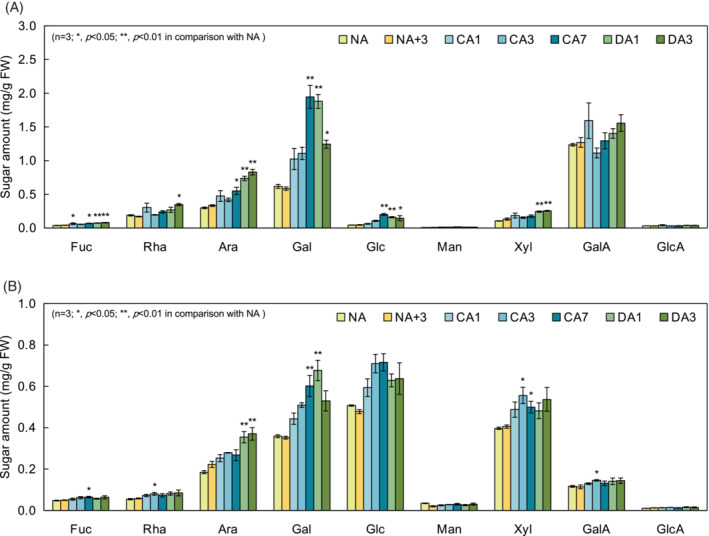
Monosaccharide composition of (A) EDTA and (B) KOH fractions determined by high‐performance anion exchange chromatography‐pulsed amperometric detection. Values represent the amount of each monosaccharide per gram fresh weight. Error bars indicate ± SEM (*n* = 3). Significant differences (Dunnett's test) between non acclimation (NA) and other treatment samples are indicated by asterisks above the bars of NA+3, cold acclimation (CA) or cold deacclimation (DA) samples, respectively (*p < 0.05, **p < 0.01).

Similarly, the EDTA fraction, which is mainly composed of pectin, showed a significant increase in Ara and Gal due to CA (Figure 6A). Pectin is composed of three major domains, homogalacturonan (HG), rhamnogalacturonan‐I (RG‐I), and rhamnogalacturonan‐II (RG‐II) (Mohnen, 2008). In these domains, Ara and Gal are found as the side chains of RG‐I, which are composed of α‐1,5‐arabinan and β‐1,4‐galactan. Interestingly, Ara and Gal increased and decreased during the DA process, respectively. On the other hand, l‐rhamnose (Rha) and d‐galacturonic acid (GalA), which constitute the main chains of HG and RG‐I, remained mostly unchanged.

The KOH fraction is mainly composed of hemicellulose, which is hydrogen bonded to cellulose microfibrils, and some pectin components that interact with cellulose (Pauly et al., 2013). Looking at the monosaccharides in this fraction (Figure 6B), Ara, Gal, Glc and d‐xylose (Xyl) were found to be abundant. Since Glc and Xyl showed increasing trends upon CA, an increase in xyloglucan was inferred. On the other hand, these monosaccharides did not fully return to NA levels in DA but maintained remained higher.

## 4 DISCUSSION

Because DA is accompanied by growth resumption as well as loss of freezing tolerance, it is necessary to examine how temperature‐induced cell wall changes differ from those during normal development if one wants to understand DA‐specific cell wall changes. We were able to confirm that the recovery of growth and changes in the cell wall during DA were caused by the increased temperature rather than the developmental stage since, based on the results of total sugar content measurements, sugar composition analysis, and PCA (Figures 3, 4, 5, 6), NA+3 plants, the control for growth‐induced changes during the DA process, turned out to be almost indistinguishable from NA plants.

The results of the PCA of the EDTA, KOH and overall fractions (Figure 4B–D) showed that the CA clusters did not move back to the NA cluster by DA treatment. It indicates that DA does not restore the composition of monosaccharides in the cell wall to the NA state, but rather that the compositional change during DA is different from the reverse of the process during CA. This differs from the changes seen in previous transcriptome and proteome analyses, where most genes and proteins simply revert to the nonacclimated state (Byun et al., 2014; Miki et al., 2018; Nakaminami et al., 2014; Pagter et al., 2017; Zuther et al., 2015). In fact, Pearson's correlation coefficients indicated that there was little significant correlation between changes in freezing tolerance (LT_50_) or growth parameters (leaf area, root length, and FW) and compositional changes in individual cell wall components during CA and DA (Table S1). Thus, in the cell wall, DA is not a simple reversion from CA to NA, and it is of interest to untangle the details, which may be important for growth arrest and resumption, for the balance between the acquisition and loss of freezing tolerance, and the response to revisiting cold.

The results of the sugar composition analysis of the soluble fraction (Figure 5A) showed that simple sugars, as represented by Glc, increased with CA, reached a maximum at CA3, and decreased with DA, which was the same as the change in total soluble sugar content and cellular osmolarity (Figures 2B and 3). This increase in osmotic concentration due to the accumulation of soluble sugars by CA treatment is important not only for preventing freezing by lowering the freezing point but also for stabilizing membranes under freezing conditions (Koster & Lynch, 1992; Levitt, 1980; Uemura et al., 2003). Changes in soluble monosaccharide metabolism may also contribute significantly to the loss of freezing tolerance in DA.

In addition to Glc, a typical monosaccharide that is accumulated under low temperatures and metabolized during DA, other monosaccharides, as represented by Ara and Gal, were increased and decreased by CA and DA, respectively (Figure 5A). Contents of Ara and Gal, which are known as the main components of the arabinogalactan (AG) sugar chains of AGPs, seem to be reflected in the Yariv reactivity of AGPs in the soluble fraction (Figure 5B). AGP is mainly recognized as an essential factor for normal cell growth (Lopez‐Hernandez et al., 2020; Shi et al., 2003; Willats & Knox, 1996; Yoshimi et al., 2020), and thus may be involved in the regulation of cell growth in CA and DA. On the other hand, AGPs may also be involved in the adaptation process to abiotic stresses, including freezing (Mareri et al., 2019). For example, fasciclin‐like AGP 4 (FLA4), also known as SOS5, has also been shown to be required for cell adhesion under salt stress (Shi et al., 2003). With respect to freezing, there is no direct evidence that AGPs are involved in freezing tolerance, but the expression of some AGPs remarkably increases at low temperatures in wheat (Faik et al., 2006). It has been suggested that the glucuronic acid residues in the glycan structures of cell wall AGPs act as calcium capacitors (Lopez‐Hernandez et al., 2020). Cytosolic calcium is an important second messenger in CA (Hiraki et al., 2019; Knight et al., 1996; Knight & Knight, 2000). Although it remains to be checked how AGP in the cell wall is involved in CA as a calcium capacitor, it stands to reason that it may be involved in controlling intracellular calcium concentration and/or in regulating the calcium cross‐linking of pectin mentioned above.

The results of the sugar composition analysis of the EDTA fraction revealed significant changes in Ara and Gal (Figure 6A). Ara and Gal in the EDTA fraction are mainly derived from arabinan and galactan, which constitute the side chains of the RG‐I region of pectin (Ebert et al., 2018; Harholt et al., 2006, 2012; Liwanag et al., 2012). This suggests that pectic arabinan and galactan contribute to the acquisition and/or loss of freezing tolerance and arrest and/or resumption of growth. It has been proposed that the accumulation of arabinan leads to a rapid recovery from the desiccated state of the resurrection plant *Myrothamnus flabellifolius* (Moore et al., 2006, 2008, 2013). This may be related to the fact that under freezing stress, plants are often subjected to both dehydration and hyperosmotic stress due to the freezing of the extracellular spaces. The percentage of Gal in the EDTA fraction (i.e., the amount of pectic galactan) correlated relatively well with LT_50_ (*r* = −0.79, Table S1). When trying to understand how galactan is involved in freezing tolerance, it is important to note that galactan accumulation is associated with the water‐binding capacity of the cell wall. It is known that in vivo truncation of galactan in RG‐I in potatoes reduces the capacity of the cell wall to retain water, presumably by affecting polysaccharide spacing and interactions in the matrix (Klaassen & Trindade, 2020). When biopolymers in the cell wall are hydrated, the water molecules involved can be divided into three categories depending on their relationship to the cell wall: free water, tightly bound nonfreezing water, and loosely bound intermediate water (Tanaka et al., 2013). The latter two are less mobile than free water and either do not freeze at all or freeze at temperatures much lower than 0°C (Bag & Valenzuela, 2017). It is quite possible that the pectic galactan‐containing cell wall network structure affects the state of bound water on the cell wall polymer surface and alters ice formation patterns. Therefore, it is important to explore the relationship between galactan and the mechanism of acquiring freezing tolerance more deeply in the future.

While both Ara and Gal increased during CA in the EDTA fraction, these monosaccharides showed contrasting changes during DA, one increasing and the other decreasing (Figure 6A). This suggests that arabinan and galactan may have different roles during DA. In the case of galactan, it has been suggested that plants cope with salt stress by accumulating galactan in roots, which inhibits plant growth (Yan et al., 2021). Therefore, plants may stop growing and/or acquire freezing tolerance by accumulating both arabinan and galactan in a coordinated manner and resume growth when galactan is degraded. There is a mechanism called reacclimation in which plants in a DA state reacquire freezing tolerance by further CA treatment. In the case of Arabidopsis, it has been suggested that plants may acquire greater freezing tolerance than that resulting from the initial CA during this reacclimation process (Byun et al., 2014). Thus, arabinan, which continues to increase during both CA and DA, may contribute to preparation for further freezing tolerance in reacclimation.

The KOH fraction exhibited large changes in Ara and Gal (Figure 6B), similar to the EDTA fraction's respective changes. The KOH fraction may contain pectin that was not fractionated in the EDTA fraction. This result may indicate that pectins with arabinan and galactan side chains interact with cellulose as well as hemicellulose. These side chains have been found to interact with cellulose microfibrils in vitro (Lin et al., 2015; Zykwinska et al., 2005, 2007), apparently in competition with hemicellulose‐cellulose interaction (Broxterman & Schols, 2018). The orchestration of cellulose with pectin and hemicellulose is believed to have a significant impact on the control of cell expansion (Cosgrove, 2014) as well as mechanical strength (Rongpipi et al., 2019). These interactions may also be inseparable from changes in bound water molecules on the cell wall surface and the control of ice formation related to structural features of the cell wall network as described above (Liu et al., 2022; Panter et al., 2020; Takahashi et al., 2021).

Besides Ara and Gal, Glc and Xyl showed increasing trends during CA. Xyloglucan, which is composed of Glc and Xyl, is the most abundant hemicellulosic polysaccharide in the primary cell wall of vascular dicots. Some involvement has been noted for xyloglucan in the low temperature response, for example, gene expression of *xyloglucan endo‐transglucosylase/hydrolase 19* (*XTH19*), which is responsible for cutting and connecting xyloglucan chains *in muro*, is induced by various stresses, including cold (Xu et al., 2020). The Arabidopsis *xth19* knockdown mutant has reduced freezing tolerance (Takahashi et al., 2020), and similar results were obtained with *xth21* (Shi et al., 2014). The functional interaction of xyloglucan with cellulose and its contribution to growth is debatable (Park & Cosgrove, 2015), given that *xxt1 xxt2*, a xyloglucan complete deletion mutant of Arabidopsis, shows only a limited phenotype in the growth of aboveground parts and roots (Park & Cosgrove, 2012; Zabotina et al., 2012). However, considering that xyloglucan accumulated during CA and did not appear to be degraded as rapidly as galactan during DA, xyloglucan would be expected to be significant in modifying the cell wall not only to help it acquire freezing tolerance during CA but also to facilitate resumption of very rapid growth during DA. Furthermore, this accumulation of xyloglucan may also be important in the “interval time” to acquire higher freezing tolerance in the reacclimation mechanism described above.

In this study, we have shown that various cell wall monosaccharides are altered by CA and DA. AGP and pectic galactan changes seemed linked to freezing tolerance and growth changes. AGPs and pectin have been shown in several studies to regulate cell growth and are also involved in plant tolerance under abiotic stress, suggesting that they contribute to the balance between the acquisition of the freezing tolerance and growth under temperature changes. By contrast, arabinan and xyloglucan did not return to NA levels after DA. This indicates that cell wall polysaccharides are altered by CA treatment, but DA treatment changes them to a specific new composition and structure that is not seen in NA or CA. Cell wall polysaccharides may work cooperatively to achieve the complex regulation required to balance the trade‐off between freezing tolerance and growth in CA and DA, and prepare for further environmental events after DA (e.g., reacclimation).

## AUTHOR CONTRIBUTIONS

Research ideas and design were completed by Toshihisa Kotake and Daisuke Takahashi. Experiments were performed and data analysis was completed by Tatsuya Kutsuno and Daisuke Takahashi. Data interpretation and manuscript writing were completed by Tatsuya Kutsuno, Sushan Chowhan, Toshihisa Kotake and Daisuke Takahashi.

## Supporting information


**Figure S1.** Scheme of cell wall fractionation.
**Figure S2.** Proportions of each fraction containing cell wall‐derived sugars.
**Table S1.** Relationships between physiological characteristic parameters of CA or DA and cell wall components.

## Data Availability

The data that support the findings of this study are available from the corresponding author upon reasonable request.
