# Supplementary material for: Temporal cell wall changes during cold acclimation and deacclimation and their potential involvement in freezing tolerance and growth
Source: Physiol Plant. 2022 Dec 29;175(1):e13837. doi: 10.1111/ppl.13837 (PMC10107845; doi:10.1111/ppl.13837)
Supplement: Supplementary file 1 — Figure S1. Scheme of cell wall fractionation. Figure S2. Proportions of each fraction containing cell wall‐derived sugars. Table S1. Relationships between physiological characteristic parameters of CA or DA and cell wall components. [file PPL-175-0-s001.pdf]

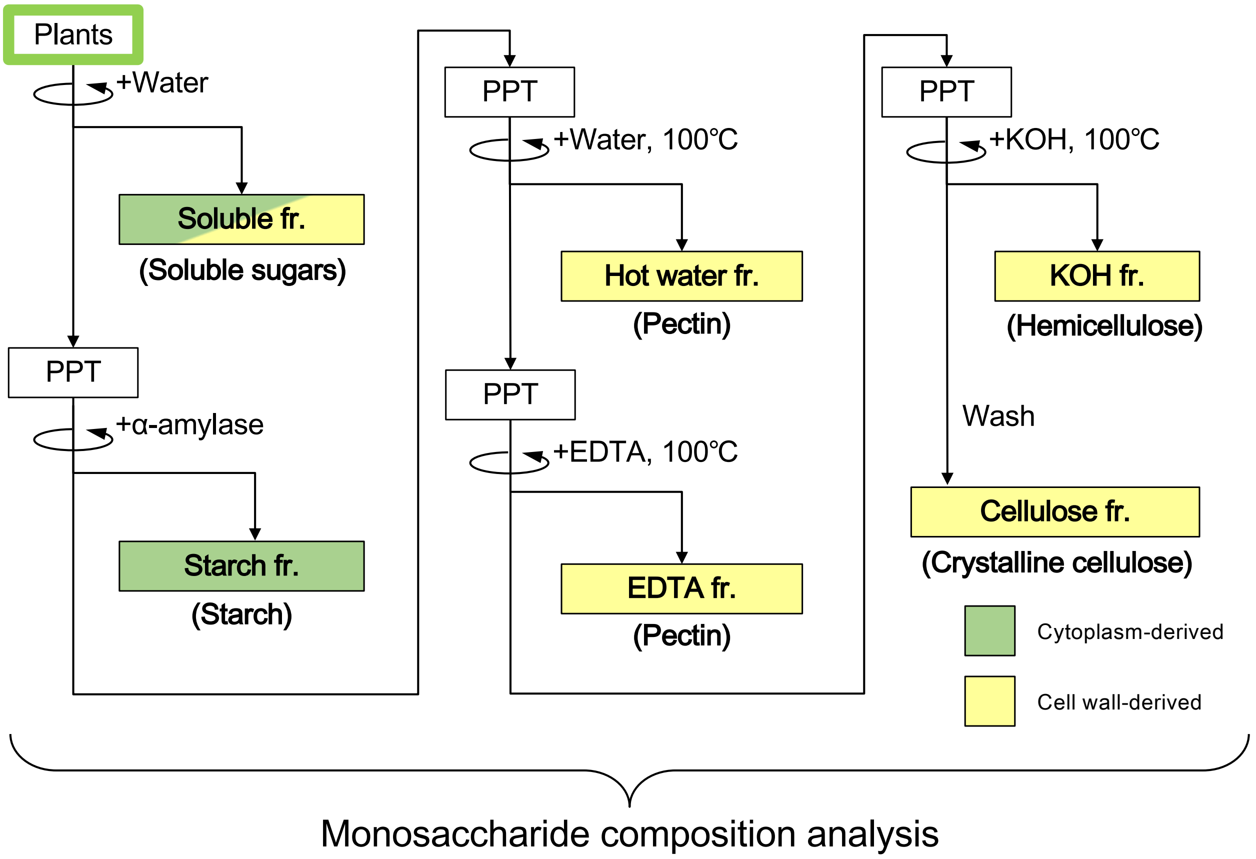

Figure S1. Scheme of cell wall fractionation. Soluble sugars, starch, pectin, and hemicellulose were successively extracted from gound arabidopsis leaves using water, α-amylase solution, hot water, EDTA solution, and KOH solution. Each fraction, except the starch fraction, contains cell wall-derived pectin and/or hemicellulose. The residue contains mainly crystalline cellulose.

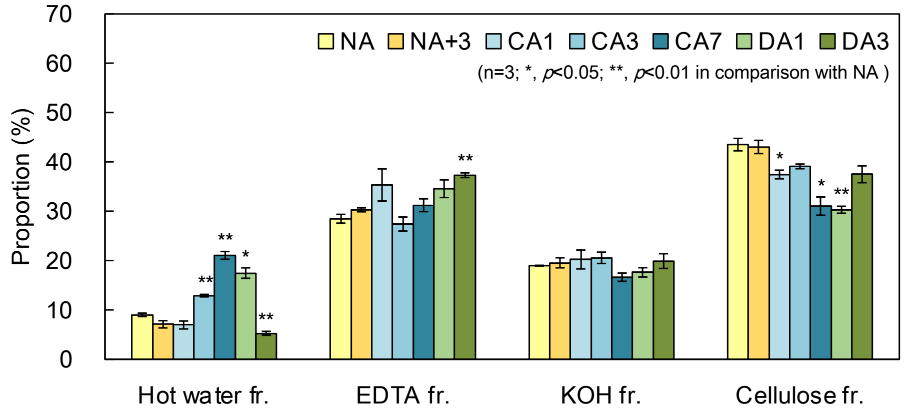

Figure S2. Proportions of each fraction containing cell wall-derived sugars. Values represent the percentage of each fraction when the total amount of hot water, EDTA, KOH, and cellulose fractions is 100. Error bars indicate ± S.E.M. (n = 3). Significant differences (Student's t-test) between non acclimation (NA) and other treatment samples are indicated by asterisks above the bars of NA+3, cold acclimation (CA) or cold deacclimation (DA) samples, respectively (*$p < 0.05$, **$p < 0.01$).

		Physiological changes					Sugar content (mg/gFW)			Proportions of cell wall fr. (%)				Soluble fraction (%)								
		LT50	Leaf area	Root length	FW	Osmolarity	Soluble fr.	Starch fr.	Total cell wall	Hot water fr.	EDTA fr.	KOH fr.	Cellulose fr.	Fuc	Rha	Ara	Gal	Glc	Man	Xyl	GalA	GlcA
Physiological changes	LT50 (°C)	1.00																				
	Leaf area (mm^2)	0.34	1.00																			
	Root length (mm)	0.75	0.81	1.00																		
	Fresh weight (mg)	-0.01	0.04	0.01	1.00																	
	Osmolarity (mOsmol)	-0.82	-0.21	-0.51	-0.25	1.00																
Sugar content (mg/gFW)	Soluble fr.	-0.95	-0.37	-0.76	-0.12	0.92	1.00															
	Starch fr.	-0.49	0.36	-0.01	0.61	0.18	0.27	1.00														
	Total cell wall	-0.55	0.19	-0.12	0.70	0.40	0.43	0.89	1.00													
Proportions of cell wall fr. (%)	Hot water fr.	-0.61	0.25	-0.21	0.44	0.19	0.38	0.94	0.74	1.00												
	EDTA fr.	-0.65	-0.32	-0.38	-0.02	0.41	0.45	0.46	0.39	0.51	1.00											
	KOH fr.	0.19	-0.11	0.04	-0.92	-0.09	-0.15	-0.64	-0.84	-0.43	0.05	1.00										
	Cellulose fr.	0.43	-0.25	0.10	-0.80	-0.09	-0.24	-0.95	-0.91	-0.88	-0.29	0.81	1.00									
Soluble fraction (%)	Fuc	-0.03	-0.41	-0.42	-0.75	0.03	0.15	-0.67	-0.79	-0.38	-0.15	0.79	0.69	1.00								
	Rha	-0.83	-0.05	-0.54	0.45	0.48	0.69	0.83	0.80	0.90	0.50	-0.54	-0.84	-0.29	1.00							
	Ara	-0.17	-0.24	-0.31	-0.86	0.12	0.19	-0.45	-0.67	-0.15	0.18	0.91	0.59	0.89	-0.17	1.00						
	Gal	-0.72	-0.43	-0.72	-0.60	0.75	0.83	-0.18	-0.13	0.05	0.29	0.41	0.26	0.66	0.31	0.68	1.00					
	Glc	0.10	0.00	0.14	0.93	-0.23	-0.21	0.49	0.65	0.24	0.07	-0.86	-0.64	-0.85	0.25	-0.93	-0.70	1.00				
	Man	0.76	0.33	0.60	-0.63	-0.50	-0.65	-0.70	-0.85	-0.66	-0.53	0.72	0.77	0.47	-0.87	0.44	-0.17	-0.56	1.00			
	Xyl	0.61	0.42	0.58	-0.73	-0.37	-0.53	-0.58	-0.79	-0.50	-0.41	0.79	0.72	0.49	-0.74	0.56	-0.04	-0.68	0.97	1.00		
	GalA	0.78	0.65	0.79	-0.44	-0.50	-0.69	-0.44	-0.61	-0.46	-0.63	0.49	0.52	0.21	-0.73	0.21	-0.33	-0.41	0.93	0.92	1.00	
	GlcA	-0.44	-0.69	-0.64	-0.70	0.56	0.56	-0.54	-0.42	-0.36	0.32	0.59	0.61	0.71	-0.10	0.70	0.84	-0.63	0.04	0.08	-0.27	1.00
Hot water (%)	Fuc	0.80	0.45	0.65	-0.36	-0.44	-0.61	-0.64	-0.65	-0.69	-0.85	0.35	0.61	0.25	-0.80	0.08	-0.29	-0.32	0.87	0.79	0.91	-0.18
	Rha	0.60	0.02	0.31	-0.63	-0.17	-0.35	-0.92	-0.82	-0.91	-0.65	0.58	0.90	0.53	-0.87	0.34	0.07	-0.52	0.83	0.74	0.70	0.32
	Ara	0.56	-0.25	0.16	-0.52	-0.15	-0.32	-0.97	-0.79	-0.99	-0.54	0.50	0.91	0.48	-0.88	0.24	0.04	-0.35	0.68	0.55	0.48	0.42
	Gal	0.42	-0.30	0.03	-0.74	-0.08	-0.20	-0.98	-0.89	-0.91	-0.39	0.73	0.99	0.70	-0.82	0.54	0.27	-0.60	0.73	0.65	0.48	0.61
	Glc	-0.39	0.33	-0.01	0.59	-0.03	0.13	0.96	0.75	0.96	0.47	-0.51	-0.91	-0.55	0.78	-0.32	-0.23	0.43	-0.60	-0.48	-0.38	-0.56
	Man	0.35	-0.20	0.18	0.29	0.01	-0.18	-0.33	0.06	-0.61	-0.30	-0.40	0.19	-0.40	-0.42	-0.63	-0.38	0.54	-0.04	-0.23	-0.06	-0.06
	Xyl	0.59	-0.25	0.16	-0.42	-0.18	-0.34	-0.94	-0.74	-0.99	-0.60	0.39	0.85	0.42	-0.86	0.14	-0.02	-0.26	0.64	0.48	0.46	0.35
	GalA	0.12	-0.43	-0.23	-0.70	0.26	0.15	-0.90	-0.69	-0.82	-0.34	0.56	0.87	0.70	-0.58	0.49	0.52	-0.60	0.46	0.40	0.22	0.75
	GlcA	0.30	-0.45	-0.13	-0.65	0.05	-0.05	-0.97	-0.79	-0.91	-0.37	0.60	0.93	0.68	-0.72	0.46	0.35	-0.52	0.56	0.47	0.30	0.68
EDTA (%)	Fuc	-0.44	0.19	0.09	0.11	0.33	0.23	0.67	0.59	0.59	0.84	-0.14	-0.46	-0.54	0.44	-0.13	-0.04	0.24	-0.43	-0.29	-0.34	-0.09
	Rha	0.77	0.14	0.40	-0.49	-0.57	-0.62	-0.80	-0.90	-0.73	-0.73	0.61	0.76	0.58	-0.84	0.39	-0.14	-0.49	0.92	0.84	0.82	0.05
	Ara	0.75	0.22	0.58	0.58	-0.63	-0.72	-0.10	0.03	-0.36	-0.55	-0.47	-0.07	-0.54	-0.44	-0.75	-0.89	0.69	0.17	-0.03	0.29	-0.67
	Gal	-0.79	0.04	-0.34	0.42	0.49	0.59	0.91	0.86	0.90	0.72	-0.50	-0.83	-0.48	0.92	-0.24	0.18	0.33	-0.84	-0.70	-0.70	-0.14
	Glc	0.46	0.17	0.24	-0.78	-0.25	-0.31	-0.72	-0.88	-0.55	-0.53	0.81	0.78	0.77	-0.65	0.70	0.24	-0.82	0.89	0.91	0.78	0.29
	Man	0.21	0.31	0.08	0.20	-0.59	-0.33	0.21	-0.13	0.39	-0.34	0.02	-0.28	0.21	0.18	0.16	-0.19	-0.11	0.17	0.19	0.27	-0.47
	Xyl	0.30	-0.71	-0.31	0.21	-0.25	-0.18	-0.59	-0.33	-0.63	-0.31	-0.11	0.35	0.18	-0.39	-0.19	-0.14	0.29	0.00	-0.23	-0.22	0.23
	GalA	0.59	-0.28	0.13	-0.43	-0.20	-0.34	-0.96	-0.76	-0.99	-0.60	0.42	0.86	0.46	-0.86	0.18	0.00	-0.28	0.65	0.49	0.45	0.37
	GlcA	0.56	-0.15	0.15	-0.72	-0.31	-0.38	-0.94	-0.98	-0.82	-0.49	0.79	0.94	0.76	-0.84	0.61	0.16	-0.66	0.86	0.79	0.64	0.44
KOH (%)	Fuc	0.45	0.47	0.56	0.82	-0.47	-0.51	0.42	0.48	0.16	-0.35	-0.76	-0.55	-0.82	0.02	-0.91	-0.88	0.83	-0.16	-0.27	0.11	-0.92
	Rha	0.76	0.43	0.63	-0.37	-0.76	-0.81	-0.38	-0.70	-0.30	-0.37	0.61	0.45	0.32	-0.65	0.37	-0.39	-0.37	0.87	0.86	0.85	-0.25
	Ara	0.51	0.03	0.07	0.36	-0.77	-0.53	-0.14	-0.29	-0.07	-0.63	-0.12	-0.07	0.18	-0.15	-0.10	-0.41	0.15	0.23	0.11	0.26	-0.45
	Gal	-0.26	0.19	0.19	0.47	0.12	0.04	0.73	0.72	0.56	0.69	-0.45	-0.62	-0.80	0.42	-0.50	-0.37	0.61	-0.53	-0.47	-0.40	-0.37
	Glc	-0.54	-0.30	-0.68	-0.38	0.51	0.68	-0.17	-0.15	0.07	-0.14	0.21	0.13	0.68	0.33	0.54	0.85	-0.62	-0.11	-0.03	-0.19	0.56
	Man	0.18	-0.08	-0.03	-0.86	0.16	0.06	-0.81	-0.78	-0.67	-0.44	0.73	0.85	0.80	-0.56	0.68	0.53	-0.85	0.68	0.70	0.52	0.61
	Xyl	0.14	-0.45	-0.35	-0.41	0.09	0.13	-0.81	-0.62	-0.70	-0.58	0.32	0.66	0.71	-0.42	0.35	0.44	-0.46	0.34	0.24	0.15	0.55
	GalA	0.49	0.03	0.26	-0.86	-0.21	-0.35	-0.84	-0.94	-0.72	-0.36	0.90	0.93	0.72	-0.80	0.70	0.21	-0.77	0.91	0.91	0.73	0.44
	GlcA	0.50	0.10	0.24	-0.68	-0.49	-0.47	-0.61	-0.90	-0.40	-0.31	0.86	0.69	0.75	-0.61	0.75	0.10	-0.73	0.86	0.88	0.71	0.21

		Hot water (%)									EDTA (%)									KOH (%)								
		Fuc	Rha	Ara	Gal	Glc	Man	Xyl	GalA	GlcA	Fuc	Rha	Ara	Gal	Glc	Man	Xyl	GalA	GlcA	Fuc	Rha	Ara	Gal	Glc	Man	Xyl	GalA	GlcA
Hot water (%)	Fuc	1.00																										
	Rha	0.86	1.00																									
	Ara	0.71	0.95	1.00																								
	Gal	0.63	0.93	0.95	1.00																							
	Glc	-0.62	-0.92	-0.98	-0.96	1.00																						
	Man	0.24	0.33	0.52	0.26	-0.49	1.00																					
	Xyl	0.72	0.93	0.99	0.91	-0.97	0.59	1.00																				
	GalA	0.46	0.83	0.87	0.92	-0.95	0.31	0.84	1.00																			
	GlcA	0.52	0.88	0.95	0.97	-0.98	0.37	0.92	0.97	1.00																		
EDTA (%)	Fuc	-0.64	-0.66	-0.64	-0.58	0.62	-0.21	-0.69	-0.58	-0.61	1.00																	
	Rha	0.89	0.87	0.76	0.77	-0.68	0.04	0.75	0.56	0.66	-0.73	1.00																
	Ara	0.44	0.19	0.27	-0.02	-0.11	0.69	0.36	-0.18	-0.02	-0.29	0.27	1.00															
	Gal	-0.86	-0.93	-0.92	-0.86	0.84	-0.35	-0.92	-0.69	-0.80	0.75	-0.94	-0.40	1.00														
	Glc	0.76	0.81	0.63	0.76	-0.62	-0.28	0.58	0.62	0.64	-0.58	0.89	-0.17	-0.76	1.00													
	Man	0.16	-0.17	-0.34	-0.27	0.41	-0.64	-0.32	-0.41	-0.37	-0.34	0.30	0.02	-0.03	0.29	1.00												
	Xyl	0.12	0.35	0.59	0.45	-0.57	0.66	0.65	0.47	0.57	-0.60	0.28	0.47	-0.52	-0.04	-0.21	1.00											
	GalA	0.71	0.93	0.99	0.92	-0.97	0.56	1.00	0.85	0.93	-0.71	0.77	0.34	-0.93	0.60	-0.29	0.67	1.00										
	GlcA	0.73	0.91	0.87	0.95	-0.85	0.06	0.83	0.79	0.87	-0.65	0.92	0.01	-0.91	0.90	0.03	0.35	0.85	1.00									
KOH (%)	Fuc	0.15	-0.25	-0.24	-0.52	0.38	0.43	-0.15	-0.60	-0.52	0.02	-0.12	0.85	0.07	-0.46	0.18	0.08	-0.17	-0.45	1.00								
	Rha	0.66	0.47	0.32	0.38	-0.18	-0.31	0.28	0.03	0.17	-0.26	0.79	0.21	-0.61	0.72	0.51	-0.15	0.30	0.62	0.02	1.00							
	Ara	0.36	0.11	0.08	0.01	0.03	-0.10	0.14	-0.13	-0.03	-0.70	0.50	0.46	-0.40	0.26	0.80	0.38	0.17	0.24	0.38	0.45	1.00						
	Gal	-0.61	-0.73	-0.65	-0.70	0.68	0.06	-0.65	-0.73	-0.71	0.91	-0.76	0.09	0.72	-0.79	-0.31	-0.37	-0.67	-0.78	0.39	-0.31	-0.49	1.00					
	Glc	-0.06	0.13	0.05	0.21	-0.20	-0.40	0.03	0.46	0.29	-0.44	0.06	-0.66	0.02	0.35	0.20	-0.02	0.05	0.20	-0.63	-0.28	0.06	-0.66	1.00				
	Man	0.63	0.86	0.76	0.87	-0.82	-0.05	0.70	0.89	0.84	-0.58	0.71	-0.31	-0.69	0.88	-0.09	0.11	0.71	0.86	-0.64	0.32	-0.04	-0.82	0.55	1.00			
	Xyl	0.47	0.73	0.76	0.77	-0.83	0.26	0.78	0.88	0.85	-0.86	0.59	-0.04	-0.68	0.58	-0.06	0.61	0.79	0.71	-0.41	0.00	0.28	-0.91	0.63	0.78	1.00		
	GalA	0.71	0.87	0.77	0.89	-0.75	-0.09	0.70	0.72	0.77	-0.43	0.85	-0.14	-0.80	0.93	-0.01	0.07	0.72	0.95	-0.53	0.68	0.04	-0.64	0.15	0.87	0.54	1.00	
	GlcA	0.59	0.61	0.47	0.64	-0.41	-0.44	0.40	0.39	0.47	-0.42	0.84	-0.17	-0.66	0.91	0.44	-0.05	0.44	0.83	-0.44	0.87	0.36	-0.61	0.16	0.66	0.35	0.87	1.00

Table S1. Relationships between physiological characteristic parameters of cold acclimation (CA) or cold deacclimation (DA) and cell wall components as expressed by Pearson's correlation coefficients. Cell wall fractions and the constituent sugar ratios of each fraction were calculated as weight percent and mol%, respectively. When correlation between each item is statistically significant (Student's t-test, *$p < 0.05$), the r-value is highlighted in yellow. FW, fresh weight; Fuc, fucose; Man, mannose; GalA, galacturonic acid; GlcA, glcuronic acid.
